# Evaluation of Myocardial Perfusion by Computed Tomography - Principles, Technical Background and Recommendations

**DOI:** 10.5935/abc.20190217

**Published:** 2019-10

**Authors:** Tiago Augusto Magalhães, Roberto Caldeira Cury, Rodrigo Julio Cerci, José Rodrigues Parga Filho, Ilan Gottlieb, Marcelo Souto Nacif, Ibraim Masciarelli Pinto, Carlos Eduardo Rochitte, Fabio Vilas-Boas, Paulo R. Schvartzman

**Affiliations:** 1 Universidade Federal do Paraná - Complexo Hospital de Clínicas (CHC) -Universidade Federal do Paraná, Curitiba, PR - Brazil; 2 Hospital do Coração (HCor) - Division of cardiovascular CT/MR, São Paulo, SP - Brazil; 3 Diagnósticos da América SA, São Paulo, SP - Brazil; 4 Quanta Diagnóstico e Terapia, Curitiba, PR - Brazil; 5 Instituto do Coração (InCor) - Universidade de São Paulo - Division of Cardiovascular CT/MR, São Paulo, SP - Brazil; 6 Casa de Saúde São José - Division of Radiology, Rio de Janeiro, RJ - Brazil; 7 Complexo Hospitalar de Niterói - Division of Radiology, Niterói, RJ - Brazil; 8 Hospital Universitário Antônio Pedro, Niterói, RJ - Brazil; 9 Instituto Dante Pazzanese de Cardiologia, São Paulo, SP - Brazil; 10 Hospital da Bahia, Salvador, BA - Brazil; 11 Hospital Moinhos de Vento - Division of Cardiovascular CT/MR, Porto Alegre, RS - Brazil

**Keywords:** Computed Tomography Angiography/methods, Myocardial Perfusion Imaging/methods, Coronary Artery Disease, Dipyridamole, Adenosine, Vasodilatation

## Abstract

Coronary computed tomography angiography (CCTA) has gained a prominent role in the evaluation of coronary artery disease. However, its anatomical nature does not allow the evaluation of the functional repercussion of coronary obstructions. It has been made possible to evaluate Myocardial computed tomography perfusion (Myocardial CTP) recently, based on myocardial contrast changes related to coronary stenoses. Several studies have validated this technique against the anatomical reference method (cardiac catheterization) and other functional methods, including myocardial perfusion scintigraphy and fractional flow reserve. The Myocardial CTP is performed in conjunction with the CCTA, a combined analysis of anatomy and function. The stress phase (with assessment of myocardial perfusion) can be performed before or after the resting phase (assessment of resting perfusion and coronary arteries), and different acquisition parameters are proposed according to the protocol and type of equipment used. Stressors used are based on coronary vasodilation (e.g. dipyridamole, adenosine). Image interpretation, similar to other perfusion assessment methods, is based on the identification and quantification of myocardial perfusion defects. The integration of both perfusion and anatomical findings is fundamental for the examination interpretation algorithm, allowing to define if the stenoses identified are hemodynamically significant and may be related to myocardial ischemia.

## Introduction

Coronary computed tomography angiography (CCTA) was introduced in clinical practice in the end of the last century to promote noninvasive visualization of coronary arteries. Its use proved it an appropriate option for the evaluation of coronary artery disease.^1-4^ Technological improvements of the equipment in recent years have allowed its application in different clinical conditions (emergency room chest pain evaluation, investigation in patients with conflicting diagnostic tests, among others). It is noteworthy that in all these conditions the background for its use rests on its high negative predictive power, making the presence of obstructive disease in the face of a negative test very unlikely.^1,2,5-8^ Hence the need for invasive coronary angiography in a large number of individuals with clinical presentation or results of noninvasive tests compatible with coronary artery disease, but without obstructive coronary disease, shows a favorable cost-benefit profile using CCTA in these clinical scenario.^1,2,5,8-10^

On the other hand, the routine use of CT scan could result in a greater number of invasive procedures, since it would show lesions without clinical manifestation and that would be submitted to interventional treatment.^11^ This limitation would stem mainly from the fact that the positive predictive value of Coronary artery CT is not as high as its negative predictive value, as it is also somewhat limited in characterizing plaques of moderate obstruction, especially when compared to other diagnostic tests.^1,4,6^ This is relevant because the correct management of patients with coronary artery obstruction requires the characterization of the functional impact of stenosis, given that atheromas that do not cause flow reduction should receive maximum clinical treatment, whereas plaques that impact myocardial perfusion could be treated with surgical or percutaneous revascularization even if it promotes a moderate decrease in vessel lumen.^12-15^

Given the importance of myocardial ischemia detection, either by echocardiography, magnetic resonance imaging, myocardial scintigraphy, or by invasive examinations that include Fractional Flow Reserve (FFR) analysis,^4,15-18^ the use of hybrid images, combining anatomical and functional findings, has become extremely desirable.^4^ However, this involves additional costs and time, which often makes this diagnostic workflow impractical. In view of the above, there was a desire to perform myocardial perfusion analyses with the MDCT itself, in the same procedure as the anatomical evaluation. This approach would involve the use of just one piece of equipment, reduced support staff, and reduced time and cost for exams. The initial attempts were made in commercially available equipment and showed favorable results, which confirmed the potential of a combined analysis, providing important data for an appropriate therapeutic management of such cases.^4,19,20^

Initial positive expectations were strengthened as technology progressed, including dual energy use, increased detector numbers, and improvements in spatial and temporal resolutions.^4,21-23^ This favorable scenario led to the development of an international multicenter trial designed to test the validity of a combined analysis of anatomy and perfusion by CT scan with the conventional way of investigating such patients, that is, angiography associated with scintigraphy.^24^ This study has shown that it is possible to perform combined CT anatomy and perfusion evaluations safely and with very favorable results.^24^

Nowadays, there are tomographs in Brazil with all characteristics necessary to ensure that such images are generated in accordance with what is described in this document. In addition, similar to what happened with the standardization of coronary angiotomography procedures by the National Agency for Supplementary Health Guidelines, the creation of a document on use is also educational and may hinder the indiscriminate use of diagnostic tests, thus avoiding the waste of resources in situations with no solid scientific evidence of the benefit these could bring.

Therefore, the purpose of this paper is to discuss in more depth the characteristics of ischemia CT research, the technological and software prerequisites, and to define which subgroups of patients would benefit from this exam.

### Physiopathological rationale of coronary tomography myocardial perfusion

Myocardial CTP is based on the principles of the theory of tracer-dilution, first developed by Stewart,^25^ in the 19th century. Images of the heart are taken during iodinated contrast injection to assess its transit through myocardial microcirculation, allowing the construction of an time-attenuation curves in the aorta and myocardium, from which myocardial blood flow (MBF) and myocardial blood volume (MBV) can be defined.^26^ Based on the principles of the theory of tracer-dilution on CT,^27^ the higher the concentration of iodinated contrast in the intravascular space and myocardial microcirculation, the greater its attenuation; and the opposite is also true. However, diffusion into the extracellular space increases over time, and after one minute its extracellular concentration is greater than the intravascular.^28^ Therefore, to obtain an accurate perfusion assessment, images must be acquired right in the beginning of the first contrast pass.

Thus, in the first-pass imaging approach, the concentration of iodinated contrast is ideally proportional to MBF in a wide range of blood flows. Areas with lower (darker) attenuation upon first contrast passage are classified as hypoperfused territories and are visually and quantitatively evaluated in comparison to adjacent myocardial territories.^26^ Another study has shown that first-pass perfusion imaging by helical CT correlates well with myocardial blood flow evaluated by microspheres.^29^ This study confirmed the feasibility of performing an atherosclerosis and MBF assessment in a single CT scan, with the possibility of quantification and semi-quantitative analysis of perfusion data by attenuation curves. These theoretical considerations were later indorsed in a human clinical study from 2012, which confirmed the accuracy of MDCT assessment to detect coronary obstructions that cause myocardial ischemia.^30^

### CT myocardial perfusion imaging - Validation

Although recently inserted in the clinical evaluation, Miocardial CTP has been subject of research for several years. In 2006, George et al.^31^ used a canine model to determine the correlation between induced epicardial stenosis and perfusion defects identified by CT, with myocardial perfusion having microspheres as reference. The favorable results encouraged further clinical studies comparing, the additional value of the combination between Coronary computed tomography angiography (CCTA) and Myocardial CTP with the use of CCTA alone. Rocha-Filho et al.^22^ showed an increase in accuracy in the combined assessment (CCTA + Myocardial CTP) compared to CCTA alone in the diagnosis of significant coronary stenosis. Adding Myocardial CTP to the strategy improved the accuracy from 0.77 to 0.90 (area under the ROC curve) in detecting stenoses.

Promising data from single-center clinical studies prompted the study CORE320.^24^ This is a multicenter study in which the combined use of CCTA + Myocardial CTP to detect flow-limiting stenoses defined by obstructions >50% associated with perfusion defects was tested, defined by the combination of myocardial perfusion scintigraphy (SPECT-MPI) and cardiac catheterization. When considering all patients, the combination protocol achieved an accuracy of 87% for the definition of disease and 93% when considering only patients with no history of previous coronary disease.

Data from this same study evaluated the performance of Miocardial CTP alone in the diagnosis of significant stenosis identified by catheterization alone, compared to MPC.^32^ The accuracy of MDCT, defined by the area under the ROC curve, was greater than that of MPC (0.78 vs. 0.69, p = 0.001), mainly due to the higher sensitivity of the first method.

Although the isolated use of MDCT for myocardial ischemia detection is not the end goal of tomography use, a recent study published by Takx et al.,^33^ described the diagnostic performance of MDCT related to other methods of myocardial ischemia analysis, taking invasive coronary FFR as a reference. In a per-patient analysis, Myocardial CTP was shown to have a 93% accuracy in detecting flow-limiting coronary stenosis, while cardiac magnetic resonance and positron emission tomography had similar accuracy (94 and 93%, respectively). These values were statistically higher when compared to methods traditionally used in myocardial ischemia analysis, such as MPC and stress echocardiography, with accuracy of 82 and 83%, respectively.

The use of dynamic myocardial perfusion in the detection of myocardial ischemia has shown encouraging results validated by different reference techniques.^26,34-39^ Clinical studies evaluating dynamic Myocardial CTP using invasive FFR as reference have shown good diagnostic performance, with sensitivity and specificity ranging from 88 to 95%, and 74 to 90%, respectively. Although promising, the evaluation of this technique has been performed mainly through small sample unicentric studies, so the potential benefits of its use in relation to static Myocardial CTP need further investigation. Similarly, the use of dual energy in the evaluation of myocardial perfusion finds favorable ground for clinical research. Recent studies have shown promising data in the attempt to detect obstructive coronary artery disease (CAD) (86-94% sensitivity and 74-98% specificity),^40,41^ however with data obtained in small samples. A prospective multicenter study to evaluate the use of this technique to identify flow-limiting coronary stenosis using invasive FFR as a reference is underway.^42^

Table 1 shows the results of selected studies evaluating Myocardial CTP performance in the search for myocardial ischemia and obstructive CAD.

**Table 1:** Evaluation of myocardial perfusion by computed tomography in the study of obstructive coronary artery disease and myocardial ischemia

Study	Year	N	Reference	Sens.	Spec.	PPV	NPV
George et al.^43^	2009	27	ICA and MPC	86	92	92	85
Rocha-Filho et al.^22^	2010	35	ICA	96	100	100	91
George et al.^30^	2012	50	MPC	72	91	81	85
Bettencourt et al.^44^	2013	101	FFR	89	83	80	90
Rochitte et al. (CORE 320)^24^	2014	381	ICA and MPC	80	74	65	86
Cury et al.^45^	2015	110	MPC	90	84	36	99
Takx et al.^33^	2015	2048	FFR	88	80	-	-
Sørgaard et al.^46^	2016	1188	MPC, MRI, ICA, FFR	85*	81*	-	-
Pontone et al.^47^	2018	100	ICA and FFR	98	54	68	96

ICA: invasive coronary angiography; MPC: myocardial perfusion scintigraphy; Spec.: specificity; FFR: fractional flow reserve; MRI: Magnetic Resonance Imaging; Sens.: sensitivity; PPV: positive predictive value; NPV: negative predictive value.

* Results of myocardial perfusion by computed tomography with MPC and MRI as reference.

### Equipment required

Any 64-channel or more (4 cm z-axis coverage) CT scan is able to perform coronary angiography, and therefore synchronizes with the electrocardiogram (ECG) and appropriate settings, being also able to study pharmacological-stress myocardial perfusion.^43-47^ For dynamic stress studies, with follow-up of the first pass of contrast through the myocardium (as opposed to a single acquisition at peak myocardial contrast - static perfusion), CT scans with at least 8 cm cover are required, either axially or in shuttle mode. Regarding image post-processing, it is recommended to use specific analysis software that allows segmenting the heart, coding the density of each area of the myocardium by color and displaying the result in a 3D map integrated with the coronary anatomy, or Bull's Eye representation form. Some newer tools allow correction of beam hardening hypo-attenuation, which is common in the inferior/inferolateral walls (from the aorta), septum (from contrast in the right ventricle) and anterior walls (from the ribs). This correction is highly recommended and can be done by probability algorithms^48^ or by dual energy spectral acquisition with reconstruction of high energy monochrome images.^49^

A contrast infusion pump, preferably with two heads, is required for dynamic injection of high flow contrast. There is no need for an infusion pump for dipyridamole, but its presence can help optimize and ensure protocol quality. Despite the safety of dipyridamole/adenosine demonstrated in past studies, emergency care supplies regularly present in radiological clinics should be readily available, as well as qualified personnel to use them. Since dipyridamole/adenosine may induce advanced atrioventricular blocks (especially in conjunction with beta-blockers), the presence of percutaneous pacemaker can be helpful. Aminophylline should be ready for infusion after dipyridamole/adenosine administration. Continuous monitoring by ECG of satisfactory quality is indispensable during infusion.

### Acquisition protocols - CT myocardial perfusion imaging

Monitoring of coronary artery tomography images, as well as CTMP, should be conducted by a specialized professional.^50^ CTMP imaging techniques vary according to manufacturer and equipment model used. Thus, we warn that for each manufacturer some adjustments should be made for protocol optimization. In addition, we emphasize that the description of patient preparation, techniques of acquisition and use of medications are suggestions based on previous studies and the authors’ experience, and may vary to meet the specific demands and workflows of each institution.

#### - Pre-exam preparation

As preparation for the exam, all patients should be fasting for at least four hours and not have caffeinated beverages in the last 24 hours.

Patients should be punctured with 18-20 gauge Jelco intravenous catheter in the antecubital vein of the right arm for administration of iodinated contrast. Another IV line in the left arm should be made for infusion of the stressor agent (dipyridamole/adenosine/regadenoson) and aminophylline as dipyridamole antagonist when necessary.

ECG, heart rate and blood pressure should be monitored by the attending physician throughout the examination. Patients with blood pressure above 100 mmHg may receive sublingual nitrate (isosorbide dinitrate [5mg] or propatylnitrate [10mg]), with a minimum interval of 20 minutes for subsequent pharmacological stress, as validated in previous safety profile studies.^24^ Although there is a theoretical anti-ischemic effect of nitrates, its use according to steps described above was not relevant to mask perfusion defects under pharmacological stress by tomography, when using SPECT-MPI associated to cardiac catheterization as a reference.^24^

#### - Use of beta-blockers as preparation for computed tomography coronary angiography

Patients may receive intravenous or oral metoprolol prior to examination. Although there is no formal guideline for this purpose, the proposal is to use the following scheme used in a previous multicenter study:^24^ If body mass index (BMI) is < 30 kg/m^2^ and heart rate (HR) is > 60 bpm, 75 mg oral metoprolol should be administered. If BMI is ≥30 kg/m^2^ and HR > 60 bpm, oral 150 mg metoprolol should be administered. If HR remains >60 bpm, intravenous metoprolol 5 mg every 5 minutes up to a total of 20 mg may be administered.

#### - Use of stressors to evaluate myocardial perfusion by computed tomography

Regardless of the mode of image acquisition or equipment available, fixed stressor administration protocols are used. In Brazil, stress protocols use mainly dipyridamole (0.56 to 0.84 mg/kg) in 4 minutes, with acquisition in the 6th minute of the beginning of injection or possibly adenosine (140 µg/kg/min for 4 minutes, with acquisition at the end of the last minute). Regadenoson can be used as a stressor at a single dose of 0.4 mg intravenous (IV) in a bolus, with stress imaging acquired within 1-2 minutes after injection.

#### 1 - 64-Row Multidetector Scanners

The assessment of myocardial perfusion at rest and stress by tomography, as well as the coronary anatomical evaluation, should always be performed in a single protocol. Aiming at a low radiation dose, we always suggest performing resting perfusion study (CCTA study itself) with the usual low-dose protocols available on the device (preferably with dose modulation and/or prospective acquisition), and the protocol under pharmacological stress whenever possible, with a slightly lower radiation dose, but diagnostic quality, always prioritizing prospective acquisition.

**Figure 1:**
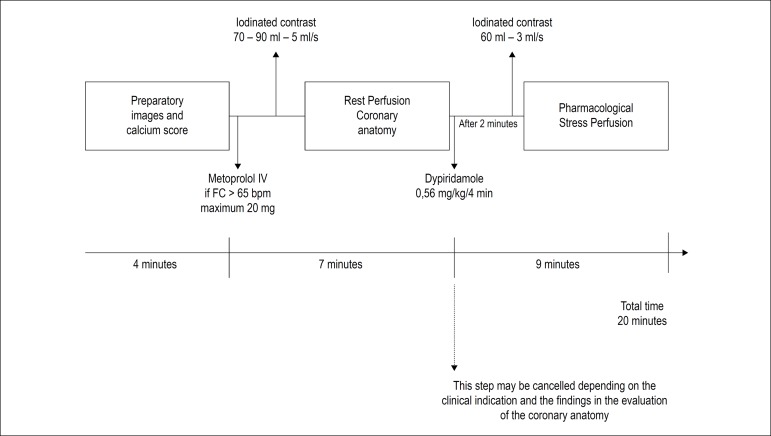
Acquisition protocol CCTA + myocardial CTP. CCTA: Coronary Computed Tomography Angiography; CTP: computed tomography perfusion.

We encourage the option of the protocol to be used (stress/rest or rest/stress) according to the experience of each center and particular characteristics of each patient or the CT scanner used. Figure 1 shows the rest/stress protocol, however stress imaging prior to rest is feasible.

As an example, we can describe the following acquisition parameters for first-generation 64-channel scanners:^19,51,52^

- Resting study - Retrospective Gating, 70-90 ml of contrast at 5 ml/s injection pump infusion after metoprolol (max. 20 mg), 64 x 0.5 mm or 32 x 0.6 mm collimation, mAs up to 850 depending on gender and weight and Kv of 100 (preferred).
- Study under pharmacological stress - Retrospective Gating, 60ml contrast at 3ml/s injection pump infusion, peak pharmacological stress acquisition, 32x1.0 mm collimation, 100 mA and 100 Kv (preferred).

Devices with latest hardware and state-of-the-art software can take advantage of available technological advances (e.g. prospective acquisition) and be based on a protocol with less radiation and the same diagnostic capability.

After iodinated contrast infusion, imaging acquisition can be performed manually as soon as the contrast is visually detected in the left atrium.

#### 2 - Volumetric acquisition scanners (eg 240-320 detectors, or high-pitch beat acquisition):

The rest protocol will simultaneously acquire anatomical image (coronary angiography) and myocardial perfusion. The start and end of acquisition should be programmed based on previously acquired calcium score images, trying to minimize the radiation dose. Acquisition parameters used include 240-320 0.5 mm detectors with 100-120 kV voltage probe, gantry rotation from 0.280 to 0.375 seconds, with prospective ECG trigger.

Intravenous contrast will be infused by pump injection in a biphasic or three-phase protocol: 100% contrast in the first phase, 30% contrast plus 70% saline in the second phase, and 100% saline in the third phase. The contrast dose will be adjusted according to the patient’s weight (see Table 2).^53^

**Table 2:** Contrast dose and flow by patient weight53

weight (Kg)	First phase: 100% Contrast (ml)	Second phase: 30% contrast and 70% Serum (ml)	Third phase: 100% serum (ml)	Flow (ml/s)
< 60	44	20	50	4
60-70	54	20	50	4.5
71-100	54	20	50	5
> 100	64	20	50	5

Modified table from George et al.^53^

Contrast injection monitoring must be performed by rapid real-time acquisitions initiated 5 seconds after the start of infusion. An apnea command is to be performed 14 seconds after the start of intravenous contrast infusion. When the contrast density peak reaches 300 UH in the descending aorta, acquisition of resting myocardial perfusion images and coronary angiography by CT will be initiated.

The pharmacological stress protocol will focus on stress myocardial perfusion image. Again, the beginning and end of the acquisition should be programmed based on previously acquired images, calcium score and rest perfusion. Acquisition parameters used include 240-320 0.5 mm detectors with 100-120 kV tube voltage, gantry rotation from 0.280 to 0.375 seconds with a prospective ECG trigger.^53^

Following administration of the stressor, a 12-lead ECG should be performed, along with blood pressure and heart rate checks.

#### 3 - Other protocols

Different techniques and forms of image acquisition are available and constantly evolving, including dual energy and dynamic acquisition. Although promising, such techniques require further investigation and radiation reduction strategies. Therefore, we will not address the protocols used for such techniques in this document.

### CCTA/CTMP Interpretation and Integration

**Figure 2:**
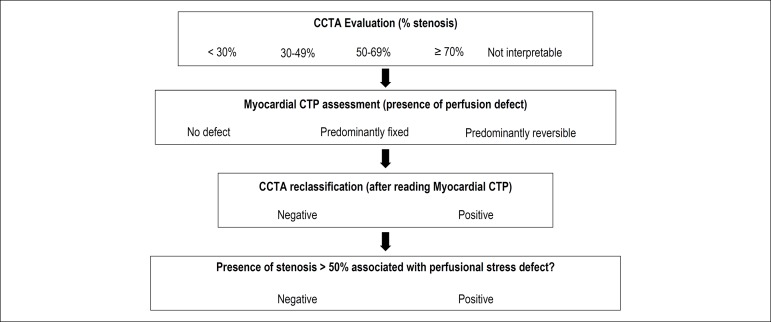
Workflow for combined CCTA + myocardial CTP analysis (modified from Magalhaes et al^57^).

The assessment of CTMP involves a sequence of steps, which must be systematized to produce a result that reflects a physiopathological change or a state of normality. In this approach, initial assessment of CCTA is recommended (Figure 2),^57^ given that the additional value of CT perfusion defects in the absence of atherosclerosis has not been investigated so far.

Once the CCTA is evaluated and any coronary stenosis and non-interpretable segments (stents, calcifications, artifacts) are quantified, the next step is to assess stress and rest myocardial perfusion. At this stage, qualitative and quantitative visual analyses (below) are used to establish the severity and extent of myocardial perfusion deficit, as well as its reversibility.

The third step in the process of image interpretation is the reclassification of anatomical findings. Due to potential limitations of luminal assessment by CCTA and the existence of intermediate stenoses,^22,51,58^ myocardial perfusion analysis may be the information required to define obstruction. In this sense, in coronary segments whose evaluation was doubtful for any reason, the presence of myocardial ischemia observed by the Myocardial CTP should strongly suggest significant stenosis.

The final step in the interpretation process is anatomical-perfusion alignment based on the integration of CCTA and Myocardial CTP findings. This process is essential to define the presence of flow-limiting stenoses,^24^ i.e. the presence of epicardial obstructions causing myocardial perfusion defects, whether fixed (fibrosis) or reversible (ischemia). This correlation should be performed mainly by multiplanar reconstructions, in order to align each epicardial vessel with its respective myocardial territory, defined by well-established myocardial segmentation models.^59^ This process should produce a correlation between epicardial stenoses and eventual perfusion defects, whose description should clear and detailed in the final report.

### Quantitative analysis of myocardial stress perfusion by tomography

Among the methods used for quantitative evaluation we can mention transmural perfusion ratio (TPR) and summed stress score (SSS), obtained through static acquisition. MBF estimates, although likely to be performed by dynamic myocardial perfusion, will not be addressed in this document.

The TPR is calculated by the average subendocardial density (in Hounsfield Units) divided by the average of the subepicardial density of each myocardial segment. This relationship showed that Myocardial CTP can detect and quantify perfusion defects compared with SPECT,^30^ and has excellent accuracy to identify perfusion defects after pharmacological stress associated with significant coronary obstruction by invasive coronary angiography.^52^ TPR less than 0.85 should be considered the cutoff value for identifying ischemic segments.^52^

Tomography SSS should be calculated based on the sum of perfusion defect of the 17 segments predefined by the American Heart Association, ranging from a scale of 0-4 for each segment (0 - normal; 1 - discrete; 2 - moderate; 3 - important and 4 - transmural perfusion defect). SSS values for ischemia quantification are: less than 4 normal, between 4 and 8 discrete, between 9 and 13 moderate, and greater than 13 important.

### Report

Myocardial CTP assessment should be divided into qualitative and quantitative analysis. The report must contain the examiner's visual and subjective impressions, followed by the quantitative assessment (TRP and SSS). The reversibility of perfusion defects is fundamental information and should be addressed in the report, as it reflects the myocardial ischemia itself.

The most important part of the report is the integration of anatomical and perfusion findings. The examiner should clearly define whether there is a correlation between luminal obstructions and perfusion defects, as well as the extent of perfusion defects and reversibility as they define the therapeutic approach.^60^

The main elements of the report are expressed in Table 3.

**Table 3:** Elements of the combined report CCTA + Myocardial CTP

Protocol used (rest-stress, stress-rest, dynamic perfusion or dual energy perfusion) and iodinated contrast volume
Scanner
Stressor agent, doses and reversing agent (when applicable)
Presence of symptoms and electrocardiographic changes in stress.
CCTA description (quantification of stenosis)
Myocardial CTP description (qualitative/quantitative analysis, artifacts)
Anatomic-perfusional integration
Conclusion

CCTA: coronary computed tomography angiography; CTP: computed tomography perfusion.

### Limitations

Myocardial perfusion study is advantageous when in conjunction with the anatomical assessment of the coronary arteries, as it benefits from the combined assessment. Thus, it is a limited strategy when considered in isolation, given exposure to ionizing radiation and iodinated contrast, which can be avoided in other diagnostic methods.

Because it uses additional doses of radiation and contrast when compared to coronary tomography alone, Myocardial CTP should be used with caution in patients with renal failure or undergoing other examinations employing ionizing radiation over a short time.

The use of vasodilatory pharmacological stress should be carefully evaluated in patients with any clinical or hemodynamic instability, as well as in patients with atrioventricular blocks, chronic obstructive pulmonary disease and asthma.

### Future perspectives

As previously mentioned, the use of dynamic perfusion by tomography allows the evaluation of myocardial iodine contrast kinetics, making it possible to quantify MBF. Additionally, the use of dual energy techniques (two x-ray tubes operating simultaneously at different voltages) allows to create an “iodine map” for the quantification of perfusion defects. Although such techniques are already available, further studies are needed to assess the impact of these approaches on clinical decision-making, as well as the increased supply of equipment that allows the use of this technique, which is still scarce in Brazil.

Recently, a new approach has emerged in the functional assessment of coronary artery disease by CT, known as CT-derived FFR. Although it uses a completely different technique, it has the same purpose of Myocardial CTP, using routine coronary tomography images without the need for pharmacological stress. Usually, coronary tomography images are transferred to a dedicated computer, where simulations based on computational fluid dynamics are performed, in order to create a three-dimensional model based on the anatomical and physiological characteristics of each patient. This model identifies stenoses and quantifies changes in intracoronary pressures, reflecting the changes found in stenoses invasively assessed by FFR.^61^ The most recent data point to an optimal accuracy of this method, and equivalent to the combined assessment CCTA + Myocardial CTP to define flow-limiting stenoses using invasive FFR as a reference.^62^ Although promising, this technique also faces limitations, such as the impossibility of use in revascularized patients with stents, as well as examinations with motion artifacts/calcification that prevent the proper identification of lumen boundaries to generate the three-dimensional model. Moreover, because it is a technique with specific and hard-availability software and hardware, FFR-CT is still restricted to some centers in the world (in Brazil, only used as a research tool).

## Conclusion

CCTA combined with stress tomography evaluation of myocardial perfusion is a safe and accurate modality for the simultaneous investigation of coronary obstructions and their repercussions on regional myocardial flow. The positive impact of this approach lies on the value added by the ischemic burden information provided by myocardial perfusion upon CT, a well-established method of anatomical and coronary stenosis assessment, with acceptable doses of radiation and the use of iodinated contrast.
